# Commentary: Musculoskeletal adverse events in dogs receiving bedinvetmab (Librela)

**DOI:** 10.3389/fvets.2025.1663398

**Published:** 2025-10-29

**Authors:** Anthony Simon, Beatriz P. Monteiro, Oliver Knesl, Adam Werts

**Affiliations:** ^1^Global Pharmacovigilance, Veterinary Medicine Research and Development, Zoetis, Parsippany, NJ, United States; ^2^Global Medical Affairs, Zoetis, Parsippany, NJ, United States; ^3^Veterinary Medicine Research and Development, Zoetis, Parsippany, NJ, United States

**Keywords:** bedinvetmab, disproportionality analyses, pharmacovigilance, Librela, rapidly progressive osteoarthritis (RPOA), accelerated joint destruction, dog, NGF

## 1 Introduction

The article “*Musculoskeletal adverse events in dogs receiving bedinvetmab (Librela)”* by Farrell et al. (1) reports an increased reporting rate of musculoskeletal adverse events (AE) in dogs treated with bedinvetmab. It is divided into three parts: a descriptive disproportionality analysis, a review of purported data entry errors and a case series of 19 clinical cases.

Bedinvetmab (Librela™, Zoetis) is a medication approved for the alleviation/treatment/control of OA-related pain in dogs. Zoetis welcomes researchers' interest in the safety of bedinvetmab and thanks the authors for raising these concerns. Zoetis does not exclude the possibility of any AE. Adverse events reported in the study are taken seriously and Zoetis continues to assess these and other reported AEs.

The present commentary highlights concerns related to the case series (Section 2) and methodology of the disproportionality analysis (Section 3), addresses allegations made regarding data entry errors (Section 4), and corrects two other identified errors (Section 5).

## 2 Selection bias and other factors in the case series that limit drawing inferences

We would like to thank Farrell at al. for publishing the case series and reporting them to Zoetis or local regulatory agencies.

Farrell et al. describe their study as a “*case-control study and case series analysis*” aimed at investigating a potential association between bedinvetmab administration and rapidly progressive osteoarthritis (RPOA). The publication discusses 19 cases of suspected joint-related AEs in dogs treated with bedinvetmab. These cases were selected by nine clinicians based on “*evidence to support a causal relationship*” and assessed by an adjudication panel using a subjective three-tiered system (“very suspicious”; “suspicious”; “insufficient evidence”). The study, however, lacks a true case-control design and scientific rigor in case selection and evaluation. Including cases from dogs with other joint diseases and chronic injuries, and blinding reviewers to treatment status, would have helped to reduce biases. Additionally, there is no control for baseline OA severity or comorbidities, and no discussion of the history of non-steroidal anti-inflammatory drug (NSAID) use (14/19 cases). Long-term NSAID use in humans is associated with accelerated OA progression (2) and RPOA (3).

The cases presented by Farrell et al., though severe, are heterogeneous, lack biological similarity, and do not consistently resemble human RPOA. Human RPOA is distinct from fast-progressing OA. It is characterized by significant radiographic joint space narrowing or subchondral bone collapse in a short period of time (6 months−1 year) (4) and features atrophic (non-bone forming) destructive arthropathy (5, 6). RPOA lacks established veterinary diagnostic criteria or pathophysiologic descriptors and has not been previously reported in dogs. In humans, OA progression varies with individuals showing “stable, “slow” or “fast” progression (7, 34). Limited data suggest similar variability in dogs, influenced by various factors; these studies did not involve bedinvetmab (8, 9).

Target animal safety studies for bedinvetmab were undertaken whereby laboratory animals received 7 monthly doses at 1X, 3X, and 10X the recommended dose. Joint risk assessments included pre-study and end-of-study radiographs of major joints, and extensive histopathological evaluation of bones and joints beyond the scope of traditional preclinical toxicology assessments. No joint risk was identified. The latter was conducted according to Good Laboratory Practices (10, 11), which are the highest regulatory laboratory safety standards globally, including auditing of the data. Nevertheless, regulatory agencies [European Medicines Agency (EMA); U.S. Food and Drug Administration (FDA)] required bedinvetmab labels to mention human RPOA as a precautionary statement, while acknowledging that this condition had not been reported in dogs (12).

Based on the available evidence, the cases do not support the existence of a canine equivalent of human RPOA, or a specific clinical syndrome associated with bedinvetmab administration.

## 3 Methodological concerns regarding the “descriptive disproportionality analysis”

Disproportionality analysis is a well-defined methodology (13) used for signal detection in large databases [e.g. Marketing Authorization Holder (MAH) databases or regulatory agencies such as EudraVigilance Veterinary (EVV)] (14) to help identify AEs with a higher-than-expected reporting frequency (15).

Disproportionality analysis most commonly involves calculation of Proportional Reporting Ratio (PRR) or Reporting Odds Ratio (ROR). Both these approaches are frequentist statistics that are inverses of each other and calculated using a 2 × 2 contingency table. The PRR compares the proportion (frequency) of reports for a specific AE [or clinical sign at the Veterinary Dictionary for Drug Related Affairs (VeDDRA) Preferred Term level] associated with the drug of interest to the proportion of reports for the same AE associated with all other drugs from a reference database. A PRR greater than 1 suggests that the AE is reported more frequently for the drug of interest compared to other drugs (or has higher odds in the case of ROR) (16). Thus, disproportionality analysis should be reported as PRR or ROR including their respective 95% confidence interval. Other disproportionality analysis methods include information component and empirical Bayes geometric mean. None of these approaches appear to have been performed in the study by Farrell et al. The authors refer to a “descriptive disproportionality analysis”, but such methodology does not appear to have been previously reported in the literature. In the abstract, disproportionality analysis seems to be referred to as “case-control study” which does not accurately reflect the nature of the study.

Farrell et al. extracted a small subset of data from the EVV database of suspected Adverse Drug Reaction Reports (EV-ADR), which is the public facing database based on EVV, and appear to have compared the number of musculoskeletal AE reports identified for bedinvetmab and other selected products. For such comparison to be valid, the datasets should be directly comparable. However, this was not the case. There are significant limitations of the datasets selected which make the analysis incomplete and invalid.

### 3.1 Data is complete for bedinvetmab

As part of the transition to new European Veterinary Medicines Regulation EU 2019/06, Zoetis took the decision to report up to three years of historical non-serious data to EVV. This is the reason for the peak in reports between January and June 2022 seen in Figure 3 of the Farrell et al. publication. The dataset is complete for bedinvetmab, but not for any other product.

### 3.2 Data is incomplete for all other products

#### 3.2.1 EV-ADR has no records of AE reports for any drug prior to 2004

Zoetis (then Pfizer Animal Health) reported all European and third country (non-European) AE reports to EVV from September 2008 onwards. Before this date, there were over 23,900 Rimadyl^®^ (carprofen) AE reports that were not reported to EVV but are available in another open-access database (OpenFDA) (17). Nevertheless, a major limitation remains that data from non-US sources (including European AEs) are not available in any open-access database prior to 2004 and is of variable completeness after that.

#### 3.2.2 Until 28 January 2022, MAHs were only obligated to submit “serious” AE case reports to EVV

At least 3,500 “non-serious” AE reports related to carprofen in the United States between 2004 and 2019 are available in OpenFDA but not in EVV. As a result, while the dataset for bedinvetmab is complete, the datasets for other products omit a substantial number of “non-serious” cases.

#### 3.2.3 Different approval dates in different regions can mean that data is incomplete

The MAHs have an obligation to submit global AE reports to EVV for all products authorized in Europe (serious cases prior to 28 January 2022 and all cases since that date). However, if a product was authorized earlier in countries outside of Europe, the respective AE reports would not be submitted to EVV. For example, Galliprant^®^ (grapriprant) was authorized in 2016 in the US and in 2018 in Europe. These cases would not have been reported to EVV.

### 3.3 Lack of access to full case details confounds analysis and findings

#### 3.3.1 Thorough deduplication is a prerequisite for disproportionality analysis

Visual inspection of the data should be considered as multiple sources may report the same incident (18, 19). Detailed review of full case information is required to identify duplicates; however, this is not possible with data extracted from EV-ADR (see below).

#### 3.3.2 EV-ADR has a limited amount of information available for a reported case

This is due to EMA”s EVV Access Policy (20) and data protection laws. For example, a very simple case in EV-ADR may contain < 20 datapoints whereas a similar case in EVV would contain >60 data points including detailed case narrative. An adequate disproportionality analysis would require full case details including the case narrative to allow for interpretation of the case (21). It is not possible to conduct an accurate disproportionality analysis using only the data available in EV-ADR.

#### 3.3.3 Disproportionality analysis is used to identify possible signals for an individual product and not directly compare different drugs

Published literature indicates that it is not appropriate to use disproportionality analysis to compare different products because they are missing incidence denominators, are subject to severe reporting bias and are not adjusted for confounding (22).

In conclusion, and based on the scientific literature on the topic, the methodology reported by Farrell et al. is inconsistent with disproportionality analysis. Referring to the methodology described in the study as disproportionality analysis does not align with established definitions of disproportionality analysis and may lead to misinterpretation of the findings. In addition, the extracted data are complete for bedinvetmab but significantly incomplete for every other product listed. It is therefore not appropriate to make any comparison or analysis between products based on the extracted data.

## 4 Misinterpretation of “translation errors” of reported cases

Farrell et al. define “translation error” as “*a clinically important discrepancy between the adverse event report (AER) submitted by the attending veterinarian and the report filed by Zoetis*”. Upon review, only one error was identified (case #10 – see Table 1 below).

**Table 1:** Review of identified “translation errors.”

**Case #**	**References**	**Translation error**	**Comment**
1	GBR-ZOETISPV-2024-UK-00209 IMAGE 1.TIFF	Wrong diagnosis	“Septic arthritis” coded. Review of the case medical records indicates that the referral veterinarian wrote to the referring veterinarian with a diagnosis of “septic arthritis”; a postmortem pathology report also stated that an infectious component could not be fully ruled out. As all clinical signs mentioned in the case narrative must be transcribed into the respective VeDDRA terms (see EVV Best Practice Guide Section 2.5 “*A complete transcription of the clinical signs mentioned in the case narrative into VeDDRA terms is very important*”) (23) and since causality is not taken into account when coding VeDDRA, septic arthritis is correctly coded as a clinical sign.
2	GBR-ZOETISPV-2023-UK-04868 IMAGE 2.TIFF	Wrong diagnosis	“Overdose” coded; “RPOA” not coded. Review of the case history provided indicates that this dog was administered 50 mg of bedinvetmab on at least one occasion which is an overdose based on reported body weight. RPOA was a diagnosis provided by the veterinarian but is not a clinical sign available for coding in VeDDRA. According to VeDDRA Guidance (24) pre-existing clinical signs are only coded if there is significant deterioration of that clinical sign reported after treatment which is why “arthritis” and “musculoskeletal disorder NOS” were coded in this situation. Coding is correct.
4	GBR-VMDDEFRA-01032/24 IMAGE 3.TIFF	Wrong diagnosis, severity and outcome	This case was initially reported to the Veterinary Medicines Directorate (VMD), supplied by the VMD to Zoetis, and then reported to EVV by Zoetis without any further editing. Investigations into this case indicate that there may have been a data connection issue between EVV and EV-ADR. The IMAGE 3.TIFF shows the outcome as “Recovered,” but if this case is currently viewed in EV-ADR the outcome is now shown as “Ongoing.” The EMA has recently confirmed to Zoetis that there was such a data connection issue which was corrected in November 2024; an appropriate warning to users is now displayed on the EV-ADR website (25).
10	GBR-VMDDEFRA-01442/24 IMAGE 4.TIFF	Wrong outcome	This case was initially reported to VMD and supplied by the VMD to Zoetis. The “outcome” was later updated by Zoetis based on new information received. In each case, there are several different fields where “outcome” is coded or described (including the case narrative). On case review, it was found that in one particular field, the necessary update was not made. This has now been corrected.
11	GBR-VMDDEFRA-01318/24 IMAGE 5.TIFF	Wrong severity, wrong outcome	This case was initially reported to the VMD, supplied by the VMD to Zoetis, and then reported to EVV by Zoetis without any further editing. Zoetis suspects that this case may have suffered from the EVV-ADR data connection issue as identified above.
13	ESP-ZOETISPV-2024-ES-00586 IMAGE 7.TIFF	Wrong diagnosis	It appears that two different cases may have been inadvertently conflated. The case number provided to Zoetis was ESP-ZOETISPV-2024-ES-00586. However, IMAGE 7.TIFF relates to a different case (ESP-ZOETISPV-2023-ES-00744). Osteosarcoma is highlighted as an incorrect diagnosis in IMAGE 7.TIFF. However, the image refers to case ESP-ZOETISPV-2023-ES-00744 involving a >13-year-old terrier which had had a left forelimb amputated as a young dog and was found to have a pathological fracture of its left femur with a histological diagnosis of osteosarcoma. Thus ESP-ZOETISPV-2023-ES-00744 is correctly coded. Case ESP-ZOETISPV-2024-ES-00586 involved a 7- to 13-year-old Staffordshire Bull Terrier (VeDDRA terms: abnormal radiograph finding, arthritis, bone and joint disorder NOS, partial lack of efficacy; Status: ongoing). These different records indicate that the two cases are distinct.
18	USA-ZOETISPV-2024-US-38409 IMAGE 9.TIFF	Wrong severity	Zoetis has reviewed this case. Based on the information provided by the reporter, the case is considered correctly coded. If additional information is provided, a follow-up submission to EVV will be made.
12	GBR-VMDDEFRA-00497/24 IMAGE 6.TIFF	Wrong diagnosis, severity and outcome	This case was initially reported to VMD and supplied by the VMD to Zoetis. “Overdose” and “bone and joint disorder NOS” were coded by the VMD. The case was later updated and additional VeDDRA terms have since been added by Zoetis based on the follow up information directly received by Zoetis. It is possible that this case may have suffered from EV-ADR data connection issue.

VeDDRA, Veterinary Dictionary for Drug Related Affairs; EVV, EudraVigilance Veterinary; RPOA, Rapidly Progressive Osteoarthritis; NOS, Not Otherwise Specified; EV-ADR, EudraVigilance Adverse Drug Reaction Report; EMA, European Medicines Agency; VMD, Veterinary Medicines Directorate.

It is important to understand that any data downloaded from EV-ADR is a snapshot in time. Pharmacovigilance data is dynamic, and cases are updated as more information becomes available to the reporting MAH. As such, discrepancies may arise if updates to case records are not reflected in the publicly accessible version at the time of data extraction. Further information regarding the relevant guidelines governing veterinary pharmacovigilance AE report data entry, including VeDDRA coding, can be found on relevant Regulatory Agency websites (14, 23–27).

## 5 Other misquotations

Farrell et al. stated “*Furthermore, despite being invited to provide annotated images to clarify this discrepancy, Zoetis declined to do so*.” However, the cited reference (28) actually states “*A summary and characterization of these incidental histopathological background findings in Beagle dogs has since been presented at the 2024 Society of Toxicologic Pathology Symposium* *(**29**). A manuscript detailing these findings is in preparation*” (28). Zoetis is actively working to ensure this information is made available in the scientific record.

Farrell et al. stated “*Notably, the joint safety claims outlined in Librela*'*s datasheet are based on radiographic assessment of five healthy beagles who received the recommended dose*.” In contrast, the cited reference (30) describes a radiographic assessment involving 24 dogs (eight in each group being administered 1X, 3X, and 10X the recommended dose) (30). It is also important to note that in the cited reference no joint risk was observed even at overdose conditions (3X and 10X), which provides additional context to the safety profile.

## 6 Conclusions

Farrell et al. conclude a strong suspicion of causality based on a “descriptive disproportionality analysis” and expert opinion reviewing cases without comparator controls. The methodology described does not align with established pharmacovigilance practices, and the conclusion exceeds the evidentiary strength of the data. The study design cannot prove/support the causation asserted by the authors. Reports of a series of cases can be useful to create awareness and inform future research, but do not allow for meaningful scientific conclusions to be drawn.

Similar concerns to those identified above have been raised in the human literature about the rapid increase in studies based on open-access pharmacovigilance databases inappropriately using disproportionality methods to compare the safety profiles of different drugs. In human medicine, guidelines address the issue of poorly reported methodology and results in published articles of disproportionality analysis (31). Furthermore, a meta-epidemiological study highlights a common issue in disproportionality studies referred to as “spin.” Spin is characterized by overinterpretation of results including inappropriate interpretations and extrapolations or misleading reporting (32). This has been described as “pharmacovigilance syndrome” (33).

The authors of this letter support increasing transparency and greater understanding of veterinary pharmacovigilance, but it is also important that publications related to veterinary pharmacovigilance follow sound scientific principles and current scientific knowledge. The publication by Farrell et al. also illustrates the need for providers of open-access veterinary pharmacovigilance databases to provide detailed explanations of potential limitations of the data available in the database and what uses of the data may be appropriate. This may help ensure that all consumers of such data are guided to avoid similar misunderstandings in the future.

Zoetis does not exclude the possibility of any AE and continues to investigate AE reports received, especially those with imaging provided, to try to identify any causal relationship between musculoskeletal AEs and bedinvetmab administration. Veterinary professionals are encouraged to report any cases they have concerns with (for bedinvetmab or any other veterinary medicine) by following national reporting requirements. Effective post-marketing pharmacovigilance relies on the collaboration of all stakeholders—animal owners, veterinary professionals, MAHs, and regulatory agencies—for the benefit of the users of veterinary medicines and the veterinary patients themselves.
